# Macro-level factors impacting geographic disparities in cancer screening

**DOI:** 10.1186/s13561-014-0013-7

**Published:** 2014-08-20

**Authors:** Lee R Mobley, Tzy-Mey Kuo, Jeffrey Traczynski, Victoria Udalova, HE Frech

**Affiliations:** School of Public Health and Andrew Young School of Policy Studies, Georgia State University, Atlanta, GA USA; Lineberger Cancer Center, University of North Carolina at Chapel Hill, Chapel Hill, NC USA; Department of Economics, University of Hawaii at Manoa, Honolulu, HI 96822 USA; Department of Economics, University of Wisconsin-Madison, Madison, WI 53706 USA; Department of Economics, University of California, Santa Barbara, CA USA

**Keywords:** Insurance regulation, Managed care spillovers, Cancer screening, Geographic disparities, Health insurance reform, Comprehensive cancer control

## Abstract

**Objectives:**

Examine how differences in state regulatory environments predict geographic disparities in the utilization of cancer screening.

**Data sources/setting:**

100% Medicare fee-for-service population data from 2001-2005 was developed as multi-year breast (BC) and colorectal cancer (CRC) screening utilization rates in each county in the US.

**Study design:**

A comprehensive set of supply and demand predictors are used in a multilevel model of county-level cancer screening utilization in the context of state regulatory markets. States dictate insurance mandates/regulations and whether alternative providers (nurse practitioners) can provide preventive care services supplied by MDs. Controlling statistically for the supply of both types of providers, we study the joint effects of two private insurance regulations: one mandating that insureds with serious or chronic health conditions may receive continuity of care from their established physician(s) after changing health insurance plans, and another mandating that external grievance review is an option for all health plan coverage/denial decisions. These private insurance plan regulations are expected to affect the degree of beneficial spillovers from managed care practices, which may have increased area-wide cancer screening rates.

**Principal findings:**

The two private insurance regulations under study were significant predictors impacted by local market conditions. Managed care spillovers in local markets were significantly associated with higher BC screening rates, but only in states lacking the two forms of regulation under study. Spillovers were significantly associated with higher CRC cancer screening rates everywhere, but much higher in the unregulated states. Area poverty dampened screening rates, but less so for CRC screening in the states with these regulations.

**Conclusions:**

Two state insurance regulations that empowered consumers with more autonomy to make informed utilization decisions varied across states, and exhibited significant associations with screening rates, which varied with the degree of managed care penetration or poverty in the state’s counties. Beneficial spillover effects from managed care practices and negative influences from area poverty are not uniform across the United States. Both variables had stronger associations with CRC than BC screening utilization, as did state regulatory variables. CRC screening by endoscopy was more subject to market and regulatory factors than BC screening.

**Electronic supplementary material:**

The online version of this article (doi:10.1186/s13561-014-0013-7) contains supplementary material, which is available to authorized users.

## Background

In the United States in 2009, breast cancer (BC) was the most common cancer in women and colorectal cancer (CRC) was the third most common cancer in both men and women [1]. Today, these facts persist and now one in four deaths in the United States is attributable to cancer [2]. Timely screening can detect or even help prevent cancer, or catch it at earlier stages and improve morbidity outcomes. Despite these benefits, the utilization of breast and colorectal cancer screening is highly variable across geography and population subgroups [3]–[7].

Numerous previous studies have examined impacts of personal and environmental factors on the utilization of cancer screening, but no studies have examined whether macro-level factors, such as the states’ insurance regulatory environments, have had an impact on cancer screening utilization. Also, it is not known whether state laws regarding permitting nurse practitioners to practice or prescribe drugs without physician oversight have impacted the cancer screening utilization climate. The major objective of this paper is to examine geographic disparities in the utilization of cancer screening among the FFS Medicare beneficiary population, and to assess whether or how differences in state health insurance and nurse practitioner regulatory environments may help to explain these disparities. We examine two state regulations that vary across the states and are expected to be correlated with the propensity to utilize cancer screening services. We expect this correlation to be stronger for CRC than for BC screening utilization, because the former is more susceptible to influence from economic factors and consumer information that can be impacted by the regulatory environment.

We adapt a conceptual model used in previous research on incidence of late-stage cancer to describe the cancer screening utilization climate [8]. We estimate an ecological model, aggregating across individuals in their counties of residence to generate county-level compositional factors reflecting the personal characteristics of the Fee-for-Service (FFS) Medicare population such as age, race or ethnicity, low income or disability status, distance to closest provider, and recent experience moving to a new residence. For the empirical models, we define these compositional factors separately for women in the FFS Medicare population 2003-2005, for use in the BC screening model. For the CRC model, the compositional variables are defined for all FFS beneficiaries during 2001-2005^a^. In both BC and CRC models, we include compositional factors and other county-level variables representing contextual factors related to both supply and demand. We have a single cross section of 3,133 counties, to which we add a second level to the model by including state-level factors describing nurse practitioner laws, shortages of medical doctors ( MDs), prevalence of MediGap insurance among persons aged 65+, state insurance market competition, and two insurance regulatory factors.

### Conceptual model

**Figure 1:**
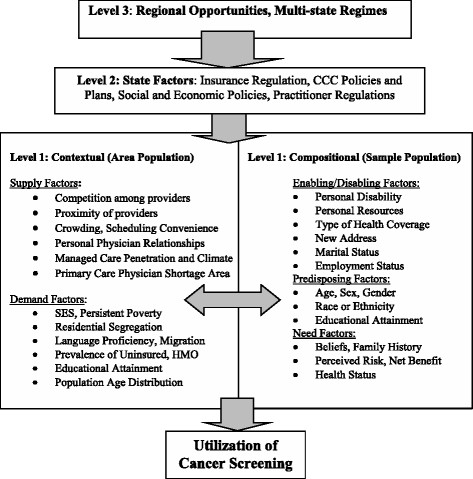
**Socio-ecological model of breast and colorectal cancer screening utilization.**

The conceptual model (Figure 1) draws from the literature and describes spatial interaction among people and characteristics of their contextual environments along the pathways to health care utilization [8]. This hybrid model incorporates the behavioral model of utilization [9] and spatial interactions in health care access and utilization [10]. The notion that interactions between race and place may influence social outcomes is not new [11], but other environmental interactions remain largely unexplored, and the broader dimension of regional opportunities has largely been overlooked in literature to date [12]. Our ecological conceptual model (Figure 1) makes explicit three levels of influence, including the county, state, and larger regional-level factors.

Each U.S. state represents a unique health care environment with unique and decentralized cancer control programs and state-specific insurance and practitioner regulations. State health care environments are governed by local and regional politics, health insurance regulation and mandates, social systems, market-level forces that determine provider and insurance supply factors, and community- or neighborhood-level forces that determine social factors. Individuals exhibit predisposing, enabling, and need characteristics, all of which interact with the forces in the broader system and yield the ecological screening rates and other compositional factors that we observe for counties.

Previous studies have found significant positive effects of participant enrollment in managed care on utilization of BC screening [13],[14], but no studies have found greater utilization of endoscopic CRC screening among managed care enrollees versus enrollees with other types of insurance. In addition to these direct effects, there is significant evidence of managed care spillovers onto other market constituents. Spillover effects from managed care have been defined and examined in the literature several ways, including changes in practice patterns, costs, or the diffusion of new technology relative to what might occur in markets with little managed care influence [15]–[17].

The economic argument for managed care spillovers is as follows. In urban areas where managed care is more prevalent, managed care plans can impact the diffusion of newer technology by directly impacting the decision of whether to invest in newer technology, or dissemination of guidelines regarding for whom it is best indicated. Managed care may also exert influence on physicians, impacting the way that medicine is practiced across all patients with a variety of insurance types. Changes in managed care provider practice patterns can spill over to people who are not insured by the managed care plans, e.g. our FFS Medicare population, but who are seen by the physicians who are affected by the information or screening guidelines the plans disseminate. Also, traditional FFS Medicare beneficiaries may compare treatment options with and be influenced by the care patterns received by their peers who are in Medicare managed care plans. Managed care plans can disseminate information and guidelines and promote preventive care services in their markets, having an impact on the way medicine is practiced or services are utilized in their markets. Managed care spillovers may impact adherence to screening guidelines by market participants, whether or not they are enrolled in managed care plans.

In our study, we have the opportunity to examine the managed care spillovers across heterogeneous markets where penetration rates vary considerably. Managed care penetration has been historically lower and later to enter in more rural areas where physicians are scarce, distances to providers are greater, and choices among insurance plans are limited. One might expect that managed care might have a greater impact on behavior in these markets, where more encouragement or guidance may be needed to convince seniors to utilize cancer screening, partly because of greater obstacles regarding access and information.

Studies find evidence suggesting that breast cancer screening rates may be higher in regions with higher managed care penetration in Medicare and the private sector [18]–[20]. As regards CRC screening, researchers examined 1999 data covering the traditional FFS Medicare population in the largest US counties and found evidence of a positive national managed care spillover associated with higher utilization of endoscopic CRC screening [21]. However, this national finding from 1999 data precedes the Medicare coverage expansion to cover endoscopy for CRC screening among persons of average risk. Another study found that national managed care spillovers were positively associated with CRC screening rates in some markets, but negatively associated with CRC screening rates in others [17]. A study that focused on endoscopy utilization for CRC screening in individual states found that managed care spillovers were significantly positive in some states in the U.S., and negative in others [22]. To sum up, the evidence regarding beneficial spillovers from managed care penetration on BC screening is consistent, however the evidence regarding spillovers on CRC screening varies across the United States, and is positive in one national study using 1999 data aggregated across all states. The study in this paper updates the data to include the period directly following the Medicare coverage expansion to cover endoscopy for CRC screening (2001-2005). The statistical modeling estimates a national managed care spillover effect on both BC and CRC screening, which is modified by local state insurance regulations through a cross-level interaction effect. This statistical approach allows the national spillover estimate to vary with local market conditions and both highlights and helps explain the spatial heterogeneity that exists from state to state in the US healthcare market.

### Health market environmental factors

Health market environment effects include both the impacts of insurance and practitioner regulations as well as spillover effects from managed care plans onto all market constituents. As regards nurse practitioners, the availability and utilization of primary care providers may affect the frequency of preventive cancer screenings. The cost of obtaining any medical procedure includes direct monetary costs, a function of individual insurance coverage, as well as indirect costs such as the travel time and other opportunity costs of visiting a primary care provider. In areas with a greater availability of primary care, the indirect costs of obtaining a cancer screening are lower, potentially leading to more screenings per year. While physicians have traditionally been the main providers of primary care services, nurse practitioners (NPs) are increasingly important in this area. In 2010, there were approximately 56,000 NPs providing primary care, representing about twenty percent of all primary care providers in the United States [23]. However, the prevalence of NPs varies widely across geography and with state regulations regarding their practice latitude.

Each state determines the range of medical services an NP can provide and the extent to which they can provide these services independent of physician oversight. Some states allow NPs to practice and prescribe independently without physician involvement, while others allow NPs to practice and prescribe only with physician collaboration or supervision. Evidence suggests that when states allow NPs to practice and prescribe independently, adults obtain routine checkups at a higher frequency and have better health outcomes [24]. Holding constant statistically the availability of MDs, we would expect these laws to affect breast and colorectal cancer screening if people in areas with laws limiting NP practice have less access to primary care, including preventive screening services. States with relaxed scope-of-practice laws for NP practice might see more cancer screenings performed as both physicians and NPs can spend more time providing patient care than in states with stricter laws.

Factors that may impact the probability of submitting to cancer screening are: information about the procedure; cost, both monetary (out of pocket copayments and deductibles, transportation) and time (participatory and travel); perceived risk from the procedure (minimal for mammography or MRI, more substantial for sigmoidoscopy and colonoscopy); and expected benefits from the procedure (early detection, *in situ* removal of potentially cancerous lesions). About 60% of seniors during this time period were enrolled in MediGap supplemental plans [25] which covered the out-of-pocket costs associated with endoscopy. All states (except Utah) mandated that insurers cover the full cost of mammography during this time period [26], so there were no out-of-pocket costs for the majority of mammograms. The time costs are much lower for mammography than endoscopy, because there is no advance preparation for mammography while preparation for endoscopy is a multi-day ordeal which includes fasting and drinking an unpleasant mixture in order to thoroughly flush and cleanse the bowel. There is a small risk of adverse effects to seniors from this bowel preparatory routine, especially to diabetics and those with restricted sodium intake or delicate electrolyte balances. There are additional risks from being put to sleep for the procedure or from accidental perforation of the bowel, and this risk is greater for colonoscopy than for the older endoscopy procedure, sigmoidoscopy. Perforation of the bowel is extremely serious and can be life-threatening. Seniors may need substantial coaching and advising by health practitioners to be willing to undergo endoscopy and then to properly adhere to the requirements for successful imaging. Travel costs for mammography are also expected to be lower than for endoscopy, simply because mammography centers are much more prevalent than gastroenterology practices or clinics that perform endoscopy. The state Comprehensive Cancer Control programs have heavily promoted the use of mammography, and most women view it as a necessary preventive health service. Endoscopy services have not been promoted as extensively, and guidelines in place at the time of this study included a portfolio of several CRC tests, including the fecal occult blood test (FOBT), which is a simpler examination of a stool sample. Because there are CRC screening options and the endoscopy test is the most costly, a greater amount of persuasion would be needed to encourage its utilization as compared to mammography, even if the expected benefits were judged to be equal. All things considered, we would expect area utilization rates of endoscopy for CRC screening to be influenced more strongly by market and regulatory factors. Supply of practitioners, distance to closest provider, managed care spillovers, and state regulations on insurance and NPs are all expected to have larger associations for CRC than for BC screening utilization rates.

## Methods

### Study population

The study population is the entire Medicare fee-for-service (FFS) population aged 65 or older and residing in the 50 U.S. states from 2001 through 2005. We used the traditional definition of Medicare FFS coverage (persons with both Parts A and B coverage). We defined a FFS Medicare cohort of persons aged 65 or older in the beginning of the study period and followed them for several years to assess whether they used screening for BC (in 2003–2005) or CRC (in 2001–2005). We used annual data from 100 percent of Medicare claims to record any mammography or endoscopy use by persons over these intervals. Persons included in the cohort remained alive, maintained coverage of Medicare Parts A and B, and remained living in the same state during the entire period. In the BC models we included only females, whereas in CRC models we included both males and females. We defined CRC screening as either sigmoidoscopy or colonoscopy utilization, and defined BC screening as either mammography or magnetic resonance imaging (MRI) utilization. While MRI images of the breast can be used for other purposes besides BC screening, we cannot differentiate these purposes. We include them here to avoid selection bias from dropping BC screening by MRI for African American women with dense breasts, for whom this BC screening procedure is recommended.

Although our study sample are FFS Medicare enrollees who are not enrolled in managed care plans, we anticipated that the market environment that influenced their utilization of preventive services would be impacted by managed care spillovers, and state insurance regulations designed to curb insurance practices that are viewed by legislators as harmful to consumers.

### Empirical specification and hypotheses to be tested

We used the conceptual model (Figure 1) to guide our choice of covariates to include in the empirical model. We chose a parsimonious set with low multicollinearity, while covering all aspects in the conceptual model. For example, it was not possible to include both educational attainment and poverty, and the lack of English language ability or percent uninsured were also highly correlated with poverty. Compositional race or ethnicity was also highly correlated with area race or ethnicity.

The dependent variables, defined as the county level screening rate for BC or CRC, were expressed as percentages. For BC, this was the percentage of the FFS female population who ever used mammography between 2003-2005. For CRC, this was the percentage of the FFS population who ever used endoscopy (colonoscopy or sigmoidoscopy) between 2001-2005.

For compositional variables, we included age groups, with the youngest age group as the reference; race or ethnicity, with whites as the reference; disability or dual eligibility status (to reflect more vulnerable populations); and degree to which the study population had recently changed residence. The aggregate proportions at the county level were expressed in units of percentages.

For contextual variables, we include the average distance to closest mammography or endoscopy provider; managed care penetration; population in poverty; and population density (defined as number of population per 1000 square miles to reflect urban extent). Managed care penetration is defined as the percentage of the insured county population enrolled in a managed care plan (Source: InterStudy). Managed care penetration of the private insurance market and poverty were expressed as percentages. Tables 1 and 2 provide sample statistics for the county-level compositional and contextual variables.

**Table 1:** **Descriptive statistics by county: mammography sample (female, 2003-2005)**

Description	Mean	Std Dev	Min	Max
Percent of the FFS Medicare sample who used mammography services in 2003-2005	55.75	7.77	7.25	93.75
Percent of the FFS Medicare sample aged 75-84	30.97	3.41	0.00	43.85
Percent of the FFS Medicare sample aged 85+	7.43	1.91	0.00	15.58
Percent of the FFS Medicare sample either with ESRD/disabled or having dual eligibility	21.14	11.07	0.00	85.88
Percent of the FFS Medicare sample who are white	86.5	12.5	2.9	100
Percent of the FFS Medicare sample who are Black	5.99	10.79	0.00	73.38
Percent of the FFS Medicare sample who are Hispanic	0.78	3.02	0.00	38.78
Percent of the FFS Medicare sample who are other races, non-white	6.70	6.57	0.00	97.06
Percent of the FFS Medicare sample who moved in 2003-2005	5.66	2.55	0.00	64.97
Averaged distance from FFS Medicare sample member residential ZIP codes to endoscopy providers in 2003-2005 (miles)	13.83	21.67	0.03	587.43
Percent of county level penetration by all managed care plans in 2001	10.00	12.47	0.00	89.50
Population density (per square mile)	0.24	1.68	0.00	68.14
Percent of the county population in poverty in 2003	13.37	4.92	0.00	36.40

**Table 2:** **Descriptive statistics by county: endoscopy sample (male and female, 2001-2005)**

Description	Mean	Std Dev	Min	Max
Percent of the FFS Medicare sample who used colonoscopy or sigmoidoscopy screening in 2001-2005	36.92	6.17	14.90	66.67
Percent of the FFS Medicare sample aged 75-84	33.23	3.42	12.77	48.08
Percent of the FFS Medicare sample aged 85+	6.70	1.68	0.00	14.77
Percent of the FFS Medicare sample either with ESRD/disabled or having dual eligibility	20.03	10.51	0.00	84.21
Percent of the FFS Medicare sample who are white	91.5	12.5	3.6	100
Percent of the FFS Medicare sample who are Black	5.98	10.81	0.00	74.95
Percent of the FFS Medicare sample who are Hispanic	0.92	3.46	0.00	43.54
Percent of the FFS Medicare sample who are other races, non-white	1.57	6.50	0.00	96.36
Percent of the FFS Medicare sample who moved in 2001-2005	9.30	6.23	0.00	98.64
Averaged distance from FFS Medicare sample member residential ZIP codes to endoscopy providers in 2001-2005 (miles)	10.2	18.1	0.07	473.64
Percent of county level penetration by all managed care plans in 2001	10.00	12.47	0.00	89.50
Population density (per square mile)	0.24	1.66	0.00	67.11
Percent of the county population in poverty in 2003	13.73	5.77	0.00	43.50

To characterize provider supply, we included the binary indicator of whether the state allowed nurse practitioners to practice independently and/or prescribe medicine as a state-level policy variable, following Traczynski and Udalova [24]^b^. We also included the state-level percentage of counties with a MD shortage.

To characterize insurance market conditions, we included the percentage of elderly persons with supplemental MediGap insurance [25]. Supplemental insurance removes out-of-pocket expenses, which can be considerable with utilization of endoscopy, especially if there are complications or follow-on screenings, coded as diagnostic rather than preventative and with much higher copays. We also included insurance industry competition measured as the market share held by the largest three private plans in the state in 2001 [27].

For insurance regulatory effects, we chose two that were expected to be especially pertinent for CRC screening by endoscopy, because of these several deterrents to utilization that existed: it was an expensive procedure, it had various guidelines in place for different screening modalities, it posed some risk to the patient and was not widely available everywhere, and it had a weak cost-effectiveness basis at the time of our study. The two regulations we study are 1) the external grievance review for health plan coverage/denial decisions (R14) and 2) the continuity of care mandate (R25) that allows enrollees who change health insurance plans to continue to receive care with established physicians who are not affiliated with their new insurance plan at time of enrollment. The external grievance review is expected to be important because many insurers at this time promoted the use of a very inexpensive stool test, which is useful in diagnosing the likely presence of cancer, but not useful in a preventative sense. (By contrast, suspicious lesions are removed during endoscopy screening which helps prevent the development of CRC). Also, once screened using endoscopy, if a lesion was found and the patient was instructed to come back for a follow-up endoscopy in the near future, this was often coded as diagnostic and not well covered by insurance. In addition, the required copayments for endoscopy varied widely across insurance plans during this time. The continuity of care mandate is expected to be important because having a relationship with an established provider is expected to help inform the patient regarding the importance of CRC screening, and also to help the patient navigate the deterrent obstacles erected by insurers who wish to avoid paying for endoscopy claims.

Managed care is expected to have a greater role in promoting cancer screening in markets where these two insurance regulations do not exist, and where consumers are more vulnerable to penurious insurance practices and less information. There are several important differences across regulated and unregulated markets which lead us to expect that the spillover effects from managed care may be larger in the unregulated markets. States enacting these laws had a greater managed care presence at baseline, were more urban, and had more accessible providers. The regulated states offer more consumer protection from insurance practices that would avoid covering costs of endoscopy, giving the consumer more leverage in health insurance coverage decisions, and the latitude to stick with established providers who can help them navigate the insurance rules. In unregulated markets, managed care penetration was later and lower at baseline, and there was a greater shortage of MDs, stricter regulation of NP practices, greater market power concentration in the health insurance industry, lower population density and higher distance to providers, and lower utilization of endoscopy, among other things (Tables 3 and 4). We expect that manage care practices that promote use of preventive care services are more necessary in these markets, and thus we would expect to see a larger effect size on the spillover estimate in the unregulated markets. In unregulated markets, managed care is expected to play a larger role in encouraging utilization because the consumer protection regulations are absent, and because markets are more sparse and transaction costs associated with obtaining information or utilizing services is greater.

**Table 3:** **Sample statistics by county (N) by regime (REG1, REG2, REG3), mammography sample (female, 2003-2005)**

	REG1^1^R14 = 1 & R25 = 1 (N = 2,056)	REG2^1^R14 = 1 & R25 = 0 (N = 632)	REG3^1^R14 = 0 & R25 = 0 (N = 445)reference group
% Mammography screening in 2003-2005, among FFS Medicare population	56.6 (7.5)	53.9 (7.6)	54.7 (8.5)
% Age 65-74 in FFS Medicare	61.3 (4.7)	63.1 (4.1)	60.8 (5.2)
% Age 75-84 in FFS Medicare	31.3 (3.4)	29.9 (3.1)	31.1 (3.6)
% Age 85+ in FFS Medicare	7.4 (1.8)	7.0 (1.8)	8.1 (2.3)
% Dual or ESRD in FFS Medicare	20.3 (10.0)	23.6 (11.7)	21.4 (14.1)
% White in FFS Medicare	87.9 (11.5)	83.1 (12.2)	85.2 (15.5)
% Black in FFS Medicare	5.2 (9.7)	7.2 (10.5)	8.0 (14.8)
% Hispanic in FFS Medicare	0.38 (1.5)	2.5 (5.8)	0.15 (0.4)
% Other race in FFS Medicare	6.6 (6.4)	7.1 (7.2)	6.6 (6.5)
% Mover in 2001-2005 in FFS Medicare	5.6 (2.6)	6.1 (2.3)	5.1 (2.3)
Average distance to closest provider	11.6 (23.1)	16.6 (18.5)	19.9 (17.1)
Managed care penetration, 2001	12.3 (13.3)	7.9 (10.8)	2.2 (4.3)
Managed care penetration, 1998	0.3 (2.1)	0.1 (0.3)	0.1 (0.1)
Population density in 2001 (in thousands)	12.6 (4.5)	15.2 (5.2)	14.1 (5.5)
% US population in poverty in 2001	47	62.5	25
% states allowing NP to practice and/or prescribe	11.8 (4.5)	15.0 (3.7)	18.1 (7.8)
% of counties with MD Shortage	63.9 (8.2)	56.9 (9.4)	65.3 (11.6)
% of Medicare population with Supplemental MediGap	63.1	64	78.5

^1^We defined three insurance regulatory ‘regimes’ based on the *combination* of the two state insurance regulatory variables (R14, external review and R25, continuity of care.

Regime 1 (REG1) was defined as having both regulations in place (R14 = 1, R25 = 1). Regime 2 (REG2) was defined as having only one of these regulations in place (R14 = 1). Regime 3 (REG3) included all other states, which had neither regulation. See Table 7 for Regime Membership by States.

**Table 4:** **Sample statistics by county (N) by regime (REG1, REG2, REG3), endoscopy sample (male and female, 2001-2005)**

	REG1^1^R14 = 1 & R25 = 1 (N = 2,056)	REG2^1^R14 = 1 & R25 = 0 (N = 632)	REG3^1^R14 = 0 & R25 = 0 (N = 445)reference group
% Screening for colorectal cancer 2001-2005, among FFS Medicare enrollees	37.8 (6.0)	35.5 (6.4)	34.8 (5.9)
% Age 65-74 in FFS Medicare	59.8 (4.6)	61.5 (4.1)	59.2 (5.0)
% Age 75-84 in FFS Medicare	33.5 (3.4)	32.2 (3.2)	33.5 (3.6)
% Age 85+ in FFS Medicare	6.7 (1.6)	6.4 (1.6)	7.3 (2.0)
% Dual or ESRD in FFS Medicare	19.3 (9.7)	22.3 (11.1)	20.1 (12.8)
% White in FFS Medicare	92.9 (11.5)	88.1 (12.6)	90.2 (15.4)
% Black in FFS Medicare	5.2 (9.7)	7.2 (10.4)	8.0 (14.9)
% Hispanic in FFS Medicare	0.4 (1.7)	3.0 (6.7)	0.2 (0.4)
% Other race in FFS Medicare	1.5 (6.3)	1.7 (7.3)	1.6 (6.1)
% Mover in 2001-2005 in FFS Medicare	9.0 (3.7)	11.2 (11.6)	8.2 (3.4)
Average distance to closest provider	8.8 (20.3)	11.9 (12.3)	14.2 (12.7)
managed care penetration, 2001	12.1 (12.5)	6.9 (9.6)	2.3 (4.1)
managed care penetration, 1998	13.6 (14.7)	8.5 (11.9)	3.4 (6.6)
Population density in 2001 (in thousands)	0.3 (2.0)	0.1 (0.3)	0.0 (0.1)
% US population in poverty in 2001	12.7 (5.3)	15.9 (6.1)	15.1 (6.4)
% states allowing NP to practice and/or prescribe	47	62.5	25
% of counties with MD Shortage	11.8 (4.5)	15.0 (3.7)	18.1 (7.8)
% of Medicare population with Supplemental MediGap	63.9 (8.2)	56.9 (9.4)	65.3 (11.6)
% Market Share Largest 3 Insurers in state, 2001	63.1	64	78.5

^1^We defined three insurance regulatory ‘regimes’ based on the *combination* of the two state insurance regulatory variables (R14, external review and R25, continuity of care.

Regime 1 (REG1) was defined as having both regulations in place (R14 = 1, R25 = 1). Regime 2 (REG2) was defined as having only one of these regulations in place (R14 = 1). Regime 3 (REG3) included all other states, which had neither regulation. See Table 7 for Regime Membership by States.

One hypothesis we can test directly is whether states with NP regulations have higher or lower cancer screening rates, or whether managed care spillovers or poverty effects exist for BC or CRC screening rates in counties. Other hypotheses we test are whether states that have adopted insurance regulations have higher or lower cancer screening rates, or whether this depends on other factors such as poverty or managed care penetration. We test whether cross-level interactions between state insurance regulations and county-level managed care plan penetration have had significant impacts on cancer screening rates. This interaction allows the managed care spillover estimate to vary with state regulatory conditions. We also test whether cross-level interactions between state insurance regulations and county-level poverty rates have significant associations with cancer screening rates. It is widely known that screening rates are lower in more impoverished communities. Whether the state regulations aimed at helping protect consumers from the cost-managing practices of insurers have different associations in the poorest communities is investigated here.

### Statistical analysis

We defined three insurance regulatory ‘regimes’ based on the *combination* of the two state insurance regulatory variables (R14, external review and R25, continuity of care). Regime 1 (REG1) was defined as having both regulations in place (R14 = 1, R25 = 1). Regime 2 (REG2) was defined as having only one of these regulations in place (R14 = 1). Regime 3 (REG3) included all other states, which had neither regulation. These multistate regimes could be considered a higher level in our model, however we did not incorporate them as such statistically, because their multilevel variance components terms were very close to zero. The two-level model was estimated using STATA XTMIXED [28] over 3133 counties in 49 states (Hawaii is excluded due to lack of data).

We estimated multilevel models using data from county and state levels after pooling the data across the 49 states. We fit random intercept models that allow state intercepts to vary. To reduce collinearity, we examined only one cross-level interaction between the county contextual and state regulation variable(s) per model. For the first model (Table 5), we interacted managed care penetration in the county with state-level regulatory regime variables. For the second model (Table 6), we included a cross-level interaction between the county proportion of population living below the poverty level and state regulatory regime variables. The estimation results for these two models (along with their baseline non-interacted models) are in Tables 5 and 6, respectively. After estimation, we use the linear model with coefficient estimates to derive the marginal effects of unit changes in the managed care or poverty variables in each of the regulatory regimes, and report these at the bottom of the tables^c^. We present post-estimation linear combination tests to assess statistical significance of these joint effect estimates presented in the bottom of Tables 5 and 6.

**Table 5:** **Ecological regression results, managed care interaction**

	BC screening, 2003-2005				CRC screening, 2001-2005			
	Base model		Interactions		Base model		Interactions	
Variable	Coeff	Pval	Coeff	Pval	Coeff	Pval	Coeff	Pval
% Age 75-84 in FFS Medicare	-0.081	0.036	-0.081	0.035	0.010	0.773	0.009	0.785
% Age 85+ in FFS Medicare	**-0.299**	**0.000**	**-0.295**	**0.000**	**-0.462**	**0.000**	**-0.455**	**0.000**
% Dual or ESRD in FFS Medicare	**-0.381**	**0.000**	**-0.383**	**0.000**	**-0.264**	**0.000**	**-0.266**	**0.000**
% Black in FFS Medicare	**0.107**	**0.000**	**0.107**	**0.000**	**0.076**	**0.000**	**0.076**	**0.000**
% Hispanic in FFS Medicare	0.038	0.404	0.036	0.420	-0.016	0.624	-0.015	0.651
% Other race in FFS Medicare	**-0.151**	**0.000**	**-0.147**	**0.000**	0.030	0.136	0.034	0.098
% Mover in 2001-2005 in FFS Medicare	**0.177**	**0.000**	**0.175**	**0.000**	**0.062**	**0.000**	**0.062**	**0.000**
Average distance to closest provider	**-0.035**	**0.000**	**-0.034**	**0.000**	**-0.040**	**0.000**	**-0.039**	**0.000**
managed care penetration	-0.016	0.131	**0.190**	**0.005**	**0.033**	**0.000**	**0.217**	**0.000**
Population density in 2001 (in thousands)	**-0.133**	**0.035**	**-0.133**	**0.034**	0.100	0.054	0.099	0.055
% US population in poverty in 2001	**-0.099**	**0.019**	**-0.092**	**0.029**	**-0.168**	**0.000**	**-0.161**	**0.000**
% states allowing NP to practice and/or prescribe	-0.109	0.935	-0.112	0.934	-0.996	0.276	-1.003	0.283
% of counties with MD Shortage	-0.204	0.134	-0.202	0.140	0.075	0.422	0.076	0.423
% of Medicare population with Supplemental MediGap	-0.088	0.230	-0.084	0.256	-0.019	0.703	-0.016	0.757
REG 1 (R14 = 1, R25 = 1)^1^	1.198	0.560	1.842	0.377	**3.771**	**0.007**	**4.379**	**0.002**
REG 2 (R14 = 1)^1^	1.358	0.578	2.024	0.415	2.370	0.155	2.824	0.102
Interaction REG1 with managed care penetration			**-0.210**	**0.002**			**-0.188**	**0.001**
Interaction REG2 with managed care penetration			**-0.213**	**0.003**			**-0.180**	**0.002**
**Sum of direct and indirect tests**			**BC Model**				**CRC Model**	
			**Joint effect**	**p-value**			**Joint effect**	**p-value**
Effects of increasing managed care penetration in REG1 states vs Unregulated states			-0.020	0.098			**0.029**	**0.003**
Effects of increasing managed care penetration in REG2 states vs Unregulated states			-0.023	0.347			0.037	0.060
Effects of increasing managed care penetration in Unregulated states			**0.190**	**0.005**			**0.217**	**0.000**

^1^We defined three insurance regulatory ‘regimes’ based on the *combination* of the two state insurance regulatory variables (R14, external review and R25, continuity of care).

Regime 1 (REG1) was defined as having both regulations in place (R14 = 1, R25 = 1). Regime 2 (REG2) was defined as having only one of these regulations in place (R14 = 1). Regime 3 (REG3) was the reference group in the estimation; it included all other states, which had neither regulation. See Table 7 for Regime Membership by States. Bold font indicates statistical significance at 95% confidence level.

**Table 6:** **Small-area regression results, poverty interaction**

	BC screening, 2003-2005				CRC screening, 2001-2005			
	Base model		Interactions		Base model		Interactions	
Variable	Coeff	Pval	Coeff	Pval	Coeff	Pval	Coeff	Pval
% Age 75-84 in FFS Medicare	-0.081	0.036	-0.074	0.052	0.010	0.773	0.004	0.914
% Age 85+ in FFS Medicare	**-0.299**	**0.000**	**-0.299**	**0.000**	**-0.462**	**0.000**	**-0.470**	**0.000**
% Dual or ESRD in FFS Medicare	**-0.381**	**0.000**	**-0.405**	**0.000**	**-0.264**	**0.000**	**-0.280**	**0.000**
% Black in FFS Medicare	**0.107**	**0.000**	**0.121**	**0.000**	**0.076**	**0.000**	**0.085**	**0.000**
% Hispanic in FFS Medicare	0.038	0.404	0.028	0.556	-0.016	0.624	-0.044	0.204
% Other race in FFS Medicare	**-0.151**	**0.000**	**-0.109**	**0.000**	0.030	0.136	**0.053**	**0.012**
% Mover in 2001-2005 in FFS Medicare	**0.177**	**0.000**	**0.160**	**0.000**	**0.062**	**0.000**	**0.054**	**0.000**
Average distance to closest provider	**-0.035**	**0.000**	**-0.036**	**0.000**	**-0.040**	**0.000**	**-0.040**	**0.000**
Managed care penetration	-0.016	0.131	-0.013	0.244	**0.033**	**0.000**	**0.038**	**0.000**
Population density in 2001 (in thousands)	**-0.133**	**0.035**	**-0.162**	**0.010**	0.100	0.054	0.094	0.069
% US population in poverty in 2001	**-0.099**	**0.019**	**-0.437**	**0.000**	**-0.168**	**0.000**	**-0.343**	**0.000**
% states allowing NP to practice and/or prescribe	-0.109	0.935	-0.432	0.749	-0.996	0.276	-1.181	0.216
% of counties with MD Shortage	-0.204	0.134	-0.166	0.225	0.075	0.422	0.092	0.346
% of Medicare population with Supplemental MediGap	-0.088	0.230	-0.083	0.260	-0.019	0.703	-0.015	0.774
REG 1 (R14 = 1, R25 = 1)^1^	1.198	0.560	-4.204	0.064	**3.771**	**0.007**	1.034	0.520
REG 2 (R14 = 1)^1^	1.358	0.578	-4.492	0.095	2.370	0.155	-1.753	0.360
Interaction REG1 with poverty			**0.434**	**0.000**			**0.207**	**0.000**
Interaction REG2 with poverty			**0.442**	**0.000**			**0.297**	**0.000**
**Sum of direct and indirect tests**			**BC Model**				**CRC Model**	
			**Joint effect**	**p-value**			**Joint effect**	**p-value**
Effects of increasing poverty in REG1 states vs Unregulated states			-0.003	0.959			**-0.136**	**0.000**
Effects of increasing poverty in REG2 states vs Unregulated states			0.005	0.939			-0.046	0.293
Effects of increasing poverty in Unregulated states			**-0.437**	**0.000**			**-0.343**	**0.000**

^1^We defined three insurance regulatory ‘regimes’ based on the *combination* of the two state insurance regulatory variables (R14, external review and R25, continuity of care).

Regime 1 (REG1) was defined as having both regulations in place (R14 = 1, R25 = 1). Regime 2 (REG2) was defined as having only one of these regulations in place (R14 = 1). Regime 3 (REG3) was the reference group in the estimation; it included all other states, which had neither regulation. See Table 7 for Regime Membership by States. Bold font indicates statistical significance at 95% confidence level.

A simplified, representative equation for both the BC and CRC models is

$$Y_{\mathrm{jk}}=\gamma_{0}+\mu_{0k}+\gamma_{1}X_{j}+\gamma_{2}C_{j}+\gamma_{3}S_{k}+\gamma_{4}S_{k}*C_{j}+e_{\mathrm{jk}+}\eta_{k},$$

Where Y_jk_ is county-level screening rate in county j and state k, γ_0_ is the overall intercept term for the 2-level model, X_j_ represents all compositional factors, C_j_ represents the contextual factors, S_k_ represents the binary indicators characterizing state insurance environments, *e*_jk_ is the residual of the outcome variable Y_jk_, and the μ_0k_ are the unique random intercepts for the state levels in the model that deviate from the overall intercept γ_0_. The residual *e*_jk_ reflects within-state variation among counties, including measurement error and variation that is not explained by the model. The state intercept term is also random and has an associated error term η_k_, which reflects variation between states beyond what is explained by the model. The variances of the two error terms are estimated among other parameters from the data. One of the main advantages of this empirical model is that the standard errors are robust to heteroskedasticity caused by variability in the size of populations across counties. The multilevel modeling accounts explicitly for the size of populations in states and stabilizes the variance for those states with small populations, producing robust estimates [29].

## Results

Tables 1 and 2 provide sample statistics for the county-level compositional and contextual variables. In Tables 3 and 4 we present sample statistics for the counties grouped into the three regimes based on insurance regulations: states with both mandates (REG1, 33 states and 2056 counties), with R14 only (REG2, 8 states and 632 counties), or no mandates (REG3, 8 states and 445 counties). Table 7 lists these three groups of states by state, with their sample counts and percentage screening for BC and CRC. It is apparent from Tables 3 and 4 that sample populations in the regulated states (REG1, REG2) are younger, more mobile, and their health markets are more competitive as regards insurance choices and more convenient as regards mammography and endoscopy procedure locations, more urban, and with higher managed care penetration than the unregulated states (REG3, 3rd column). Average managed care penetration in 1998 and in 2001 was higher in the regulated states than in the unregulated states, and the average penetration rate fell over 1998-2001 in all areas. Unregulated states also had lower average endoscopy utilization, greater shortages of MDs, stricter regulation of NP practices, more concentrated/less competitive health insurance markets, were more rural, and had greater average distance to cancer screening locations. Prevalence of supplemental MediGap coverage was higher in the unregulated states.

**Table 7:** **Sample statistics by state and regime**

State name	Mammography screening cohort		Colorectal screening cohort	
	N	% Screening	N	% Screening
**Total all groups**	**12,691,175**	**58.0**	**17,249,117**	**40.1**
Total Group 1, REG1: (R14 = 1, R25 = 1), 33 states	9,784,064	58.0	13,279,641	40.5
Alaska	15,220	56.2	22,585	37.9
Arizona	169,155	62.5	223,305	39.6
Arkansas	165,838	54.8	224,275	35.4
California	880,322	57.3	1,126,335	38.2
Colorado	119,296	58.7	156,466	39.7
Delaware	46,003	63	64,072	48.4
Florida	858,753	64.2	1,139,258	46.3
Illinois	612,065	55	818,437	37.8
Indiana	337,409	55.8	471,278	37.9
Iowa	190,402	59.6	274,939	39.5
Kansas	152,815	61	211,602	39.2
Kentucky	222,701	55.2	307,484	38
Louisiana	188,609	56.8	244,130	37.6
Maine	84,136	66.9	121,387	43.1
Maryland	248,969	58.9	346,573	44.8
Massachusetts	286,348	62.2	362,711	40.4
Michigan	556,834	62.6	765,461	43.1
Minnesota	214,559	63.1	314,019	43.9
Missouri	283,628	55.6	387,278	39.3
New Hampshire	67,023	63.5	95,298	41.4
New Jersey	434,880	50.9	567,836	39.6
New York	753,806	55.3	1,040,451	40.3
North Carolina	430,080	60.4	587,505	42.6
Oklahoma	179,423	54.5	248,870	35.9
Oregon	108,850	61.9	151,816	37.8
Pennsylvania	601,984	55.1	819,431	38.1
Rhode Island	37,545	58.6	50,326	41.7
South Carolina	223,371	60.1	308,796	42.5
Tennessee	286,680	55.2	395,590	38.1
Vermont	35,362	61.8	50,631	40.4
Virginia	352,984	57.4	492,814	42.4
Washington	229,864	61.6	314,345	41
West Virginia	114,593	58	162,307	36.8
Wisconsin	294,557	60	412,030	42.3
Total Group 2, REG2: (R14 = 1, R25 = 0), 8 states	2,184,838	57.2	2,961,963	39.1
Connecticut	195,009	60.9	245,186	42.7
Georgia	345,422	58.3	464,828	40.6
Hawaii	39,234	56.9	56,573	39.6
Montana	53,457	62.1	79,539	39.8
New Mexico	69,558	54.1	100,328	34.2
Ohio	568,259	58.4	783,948	39.3
Texas	836,386	54.8	1,118,495	37.6
Utah	77,513	57.6	113,066	41.8
Total Group 3, REG3: (R14 = 0, R25 = 0), 8 states	722,273	57.2	1,007,513	37.8
Alabama	234,244	59.3	319,335	39.2
Idaho	57,586	56.6	82,703	36.7
Mississippi	156,305	51.3	211,398	36.4
Nebraska	100,748	56.6	146,001	36.1
Nevada	56,113	56.7	75,709	35.6
North Dakota	42,275	64.1	62,867	41.5
South Dakota	49,723	62	72,116	40
Wyoming	25,279	57.7	37,384	35.3

In Tables 5 and 6, we provide the empirical results from multilevel modeling of ecological factors at the county level with state-level factors as higher levels of influence. There was considerable variation across counties reflecting both socio-demographic and market-related factors. Endoscopy services spread unevenly across Medicare markets during this period, resulting in some counties with no services requiring long travel distances to reach the closest provider [17]. Average distance to closest provider was a significant and negative predictor for both types of screening. States varied widely in their regulation of nurse practitioners, but these regulations had no significant associations in our models. State-level MD shortage and prevalence of supplemental MediGap insurance were not statistically significant predictors. State insurance market competition was statistically significant, but did not change other effect estimates with the exception of population density, which became insignificant when this insurance competition variable was added to the model. Keeping population density in the model is important, because managed care penetration is higher in more urban markets and we want to control for the urbanicity aspects. For parsimony, we dropped the state insurance market competition variable in the specification presented in Tables 5 and 6.

Penetration of managed care insurance and poverty rates varied widely across the counties and states, which empowers statistical tests of whether the prevalence of managed care market spillover effects or poverty were associated with higher or lower utilization of cancer screening, and whether these associations varied across states with different insurance regulatory regimes. In Tables 5 and 6, the column labeled “base model” reports effect estimates when no interaction terms are included in the model. The column labeled “interactions” reports the effect estimates when interaction terms are included in the model. In models with no interaction effects included, the state regulatory variables are only statistically significant predictors for CRC screening, where the rate is about 3.77 percent higher in states with both regulations (REG1) relative to unregulated states (REG3). County poverty rate is a significant negative predictor, and the effect size is almost twice as large for CRC as for BC screening. Managed care spillover effects are not significant for BC screening utilization rates, but they are significant and positive for CRC screening rates in counties. This positive national effect estimate is consistent with the previous study which estimated it for a more dated FFS Medicare population [21]. State NP regulations are not significant predictors in these models.

In models with interaction effects, the assessment of statistical associations is done using combined direct and indirect effect estimates, along with joint tests of their significance. At the bottom of Table 5, we report the change in BC or CRC screening rates for a unit (percent) increase in the managed care penetration rate in the different regulatory regimes, and provide a joint test of the statistical significance of each scenario. At the bottom of Table 6, we report the change in BC or CRC screening rates for a unit (percent) increase in the poverty rate in the different regulatory regimes, and provide a joint test of the statistical significance of each scenario.

In the interaction models presented in Table 5, we found that managed care spillovers were significant and positively associated with BC screening rates, but only in the *unregulated* states. In the unregulated states, a 1% increase in managed care penetration would be associated with about a 0.19% increase in county BC screening rate. Managed care spillovers were significant and positive for CRC cancer screening rates everywhere, but much larger in the unregulated states. In the most heavily regulated states (REG1), a 1% increase in managed care penetration was associated with 0.03% increase in county CRC rates, while in the unregulated states the association was about 0.22% higher screening rate. For the groups of states with only one regulation (REG2), the effect seems a little larger but is only weakly significant (p-val 0.06).

In the interaction models presented in Table 6, we found that area poverty rates were significant and negatively associated with BC screening rates, but only in the *unregulated* states. In the unregulated states, a 1% higher poverty rate was associated with about a 0.44% increase in county BC screening rate. For CRC screening, poverty associations were negative everywhere but the dampening effect of poverty on screening was higher in the unregulated states. For example, a 1% higher poverty rate in the most regulated states (REG1) was associated with only about 0.14% lower screening, while in unregulated states (REG3) it was about 0.34% lower screening rate. These differences across the most regulated (REG1) and unregulated (REG3) states were highly significant.

## Discussion

There are dozens of state insurance regulations aimed at curbing insurance practices that were considered by legislators to be harmful to consumers, that emerged during the latter nineties when there was a general backlash against managed care [26],[30],[31]. We initially considered ten regulations that have been studied in previous literature that examined economic outcomes such as increases in premiums and reductions in affordability of insurance and higher rates of uninsurance [31]–[34]. These studies examined health plan liability laws and direct access to specialist mandates [31]; community rating, any willing provider, and guaranteed issue laws [35]; community rating by health status or age, and high risk pools [32]; and external grievance review for health plan coverage decisions [25]. While these economic outcomes are important, no studies have examined whether state-level variation in health insurance regulatory environments have impacted aggregate health prevention behaviors, a perspective that is critically important as our nation grapples with healthcare reforms [36]. The continuity of care law was found influential in the only health outcomes study to date in this literature, which looked at late-stage CRC cancer outcomes [8].

We chose to examine two regulations that were expected to be especially important for preventive care services utilization outcomes. These are the external grievance review for health plan coverage/denial decisions (R14) and the continuity of care mandate (R25) that allows enrollees who change insurance plans to continue to receive care with established physicians who are not affiliated with their new insurance plan at time of enrollment. We expected both of these regulations to be significant predictors because they both empower consumers to contest coverage denial decisions by insurers or higher copays for some services as compared to others, and enable better informed decisions by uniting consumers with their established physician to help in making these choices and demands. Both of these regulations proved to be significant predictors in the models, and interacted significantly with local area conditions such as poverty or managed care penetration to predict area screening rates. The regulatory effects were stronger for the riskier, more expensive and more controversial CRC screening by endoscopy than for BC screening by mammography. This finding lends credence to our theory that these regulations empowered consumers to make more autonomous, better informed decisions regarding utilization of insured preventive care services.

Because evidence suggests that managed care has seemed to promote cancer screening directly among enrollees and indirectly as national spillover effects in more penetrated markets, an interesting question is whether these beneficial managed care spillovers are smaller or larger in the dozens of states that have enacted regulations of private insurance practices. Our theory is that health insurance regulations that impact the ability of consumers to choose particular services have downstream impacts on utilization of preventive services in the market. The role that managed care plans play in shaping market outcomes stems from their dissemination of practice guidelines and protocols, which help shape the way that medicine is practiced and which services are offered in their markets. We find a small national spillover for endoscopy utilization in the non-interacted models which restrict it to be the same everywhere, consisted with an earlier study published using older FFS Medicare data [21]. However, as suggested by other studies [17],[22], the magnitude of these managed care spillovers was expected to vary across states. We tested whether these spillovers varied across the regulated versus the unregulated states, holding constant statistically other market factors that varied considerably across these groups of states. We found that managed care spillovers were significantly higher in the unregulated markets. Another hypothesis we tested through the interactions was whether the negative impact of poverty varied across the regulated and unregulated states. We expected that this negative association would diminish in regulated versus unregulated states, if these regulations benefited poorer constituents. We found statistically significant evidence that the negative impact of poverty was lower in the regulated versus the unregulated states.

## Conclusions

Observed geographic disparities in breast and colorectal cancer screening rates reflect ecological differences in compositional, contextual, and regulatory factors among health markets. State regulations which curbed nurse practitioner services were not a significant predictor in either of the cancer screening utilization models. However, two state insurance regulations that empowered consumers with more autonomy to make informed utilization decisions exhibited significant associations with screening rates, which varied with the degree of managed care penetration or poverty in the state’s counties.

Managed care penetration has been historically slower and lower in more rural areas where physicians are scarce and distances to providers are greater. We expected that managed care might have a greater association with utilization behavior in these more rural, emerging managed care markets, where more encouragement or guidance may be needed to convince seniors to utilize cancer screening, partly because of greater obstacles regarding access. These more rural conditions are prevalent in states where the two regulations we study do not exist to help seniors navigate or counter the reluctance of providers to cover or promote expensive new screening technologies. In these more rural, unregulated states, the role of managed care spillovers encouraging screening utilization was expected to be greater, and we found evidence to support this expectation. In unregulated markets, we found that the managed care spillovers were greater than in the regulated markets, and that these associations were stronger for CRC than for BC screening. CRC screening by endoscopy is the more expensive, controversial screening modality so this finding is not surprising. Encouragement to utilize endoscopy for CRC screening needs to overcome transaction costs associated with both time and distance, lack of information, uncertainties regarding risk from the procedure, and complicated screening guidelines and reimbursement schedules.

The cross-level interactions between managed care spillovers and regulations were small in comparison to the cross-level interactions of state regulatory regimes with poverty. We find that poverty dampens screening rates less in the regulated states than in the unregulated states, *ceteris paribus*. Thus the regulated states seem to have poorer communities that are better informed, empowered, or better motivated to utilize CRC screening than their counterparts in unregulated states.

Finally, our findings suggest that during this time period, utilization of endosopy services for CRC screening was more subject to influence by market factors than utilization of mammography for BC screening. Compared to mammography, endoscopy posed greater risk, is more unpleasant, required copayment of substantial out-of-pocket costs, and was often denied coverage for repeat tests recommended by physicians as follow-up procedures. Thus it is not surprising that we find that the influence of both area poverty and managed care spillovers on cancer screening rates is more substantial for CRC screening by endoscopy than for the simpler, less expensive and less controversial BC screening.

## Endnotes

^a^The period of study, 2001-2005, is rather dated. However, this study is unique and was only possible with financial support from the National Institutes of Health. At the time the study began, obtaining 100% abstracts of FFS Medicare claims, to include all breast and colorectal cancer screening utilization events for five consecutive years, was financially feasible. Now, the cost of these data is about five times higher and it is much more difficult to obtain permission for researchers to access 100% of the Medicare claims extracts. Because these data are based on 100% FFS Medicare population data, they are perfectly generalizable for the vast majority of elderly persons, for whom these cancers are most prevalent. They do provide a useful snapshot for market-driven health systems of the impact of insurance and other regulations on an important preventive health outcome – cancer screening. Thus, the data used here may be dated, but they are uniquely valuable, and we will share them with other researchers at no cost.

^b^Following Traczynski and Udalova (2013), we define NP independent practice authority as the absence of statutory or regulatory requirements for physician collaboration, delegation, direction, or supervision. Independent prescriptive authority is defined as the ability of NPs to prescribe medications (including controlled substances, if allowed) independent of physician collaboration, delegation, direction, or supervision.

^c^To see how the marginal effects are calculated for the different regime interaction terms, suppose

$$Y=\ldots +\gamma_{2}C+\gamma_{3}S_{1}+\gamma_{4}S_{2}+\gamma_{5}S_{1}*C+\gamma_{5}S_{1}*C_{....}$$

Where:

Y is screening rate in county

C is managed care penetration

S_1_ = 1 when REG1 = 1, otherwise zero

S_2_ = 1 when REG2 = 1, otherwise zero

S_3_ = 1 is the omitted reference regime, when S_1_ = 0 and S_2_ = 0

Then:

∂Y/∂C = γ_2_ + γ_3,_ when S_1_ = 1

∂Y/∂C = γ_2_ + γ_4,_ when S_2_ = 1

∂Y/∂C = γ_2,_ when S_1_ = 0 and S_2_ = 0

## Authors’ original submitted files for images

Below are the links to the authors’ original submitted files for images. Authors’ original file for figure 1

## Abbreviations

BC: Breast cancer
CRC: Colorectal cancer
FFS: Fee-for-service
MD: Medical doctor
NP: Nurse practitioner
