# Supplementary figures and images for: Macro-level factors impacting geographic disparities in cancer screening
Source: Health Econ Rev. 2014 Aug 20;4:13. doi: 10.1186/s13561-014-0013-7 (PMC4883991; doi:10.1186/s13561-014-0013-7)

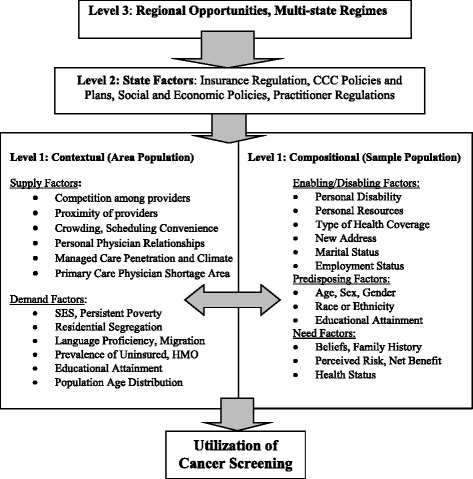

Supplement: Supplementary file 1 — Authors’ original file for figure 1 [file 13561_2014_13_MOESM1_ESM.gif]
